# ‘Back to Basics’: A Self‐Administered Survey of Undergraduate Dentists' Prevalence, Impact and Understanding of Lower Back Pain

**DOI:** 10.1111/eje.70035

**Published:** 2025-08-25

**Authors:** Samuel Cope, Elizabeth Willasey, Daniel Dadnam, Laura Gartshore

**Affiliations:** ^1^ School of Dentistry University of Liverpool Liverpool UK

**Keywords:** dental education, dental student, ergonomics, lower back pain, musculoskeletal disorders, occupational health

## Abstract

**Introduction:**

The aim of this paper is to assess the prevalence, impact, and understanding of Lower Back Pain (LBP) amongst final year dental students to inform the development of a module in the dental curriculum. LBP has a significant impact on the dental workforce; yet little is reported about the profession's knowledge, or the necessity for tailored self‐care education.

**Materials and Methods:**

A single‐centred online survey was administered to final year dental students at a UK dental school. This used carefully selected criteria following the review of the limited validated evidence base, with a robust methodological approach to survey design.

**Results:**

There was an 86% response rate. LBP is prevalent amongst dental students, with 58% experiencing LBP in the past 6 months. 29% had reduced recreational and professional activity as a result, and 14% had sought professional help. Understanding of LBP varied; 92% identified the practice of dentistry to be the greatest contributing factor, whilst 89% deemed correcting posture to be the most effective prevention method. A majority, 53%, believed LBP would resolve without treatment, although confidence in management was poor. Almost all responders supported inclusion of back pain education in undergraduate dental curricula.

**Conclusion:**

This research has revealed a belief system that may have negative influences on dental professionals' lower back health. Dental professionals may lack education on musculoskeletal disorders and have a poor understanding of the prevention and management of LBP. The results have informed the development of a LBP module aiming to improve undergraduate awareness and positively impact future working life.

## 1 Introduction

Musculoskeletal pain is ubiquitous amongst dental professionals, with a reported lifetime occurrence affecting 64%–93% of the workforce [1]. LBP is the most frequent dental musculoskeletal complaint leading to absenteeism, medical care seeking, premature retirement, and impacting on quality of life [2, 3]. It is postulated that dental professionals are reported to experience LBP more frequently than other members of the public. This has been suggested to be a result of prolonged hours in sedentary stationary positions, poor posture, lack of exercise outside of work, repetitive movements, stress, lack of ergonomic advice, and education on musculoskeletal disorders coupled with a poor understanding of the prevention and management of LBP [4, 5, 6, 7]. Evidence suggests that females report greater pain intensity and prevalence pain scores for LBP on visual analogue scales (VAS) [4, 8, 9, 10].

An epidemiological survey of 398 undergraduate dentists found 54% had experienced LBP, indicating that for many practitioners, LBP starts during university years. A further recent study conducted in Germany corroborated to show that the presence of musculoskeletal pain, especially in the back area, was found to be higher in dental students than in the general population [11]. The prevalence and pain intensity of LBP (0% equating to no pain and 100% being a most painful experience) amongst dental students has been reported to peak during the final year of undergraduate training. However, undergraduates' knowledge of LBP is poorly understood [4, 5, 7, 8].

Despite the available research concerning the prevalence of LBP in dental undergraduates, there is an evident gap in the literature base in relation to the impact of LBP for dental undergraduates on both recreational and professional activities and understanding of causes and management strategies, which this study will aim to address. Moreover, a recent umbrella review also highlighted the low‐quality available evidence into the prevalence of musculoskeletal disorders in dental professionals and highlighted the need for further research with robust methodological design [12].

## 2 Prevention and Management of LBP

Understanding the prevention and management of LBP is very important, particularly for clinicians who have experienced LBP either acutely or chronically. Evidence suggests that unhelpful beliefs regarding posture and exercise can lead to greater levels of disability and may lead to future chronic and persistent pain [13, 14, 15]. Table 1 documents risk predictors of lower back pain. Prevention of LBP should take a biopsychosocial approach, as physical, psychological, and social factors can influence risk, which can be modified, for example, through smoking cessation, a healthy diet, increased physical activity, and participation in stress management techniques. Acute LBP responds within a shorter timeframe and has less likelihood of recurrence when sufferers remain active, limit bed rest, take non‐steroidal anti‐inflammatory drugs, and use methods to change the stimulus to the area, such as superficial heat or self‐massage techniques that can be done at home or professionally [16]. Table 2 shows recommended management strategies for acute LBP. Chronic LBP (> 12 weeks) is managed somewhat differently (as evidenced in Table 3); hence, the management of persistent LBP requires greater emphasis on psychosocial management, such as exercise therapy, CBT, and mindfulness‐stress reduction [16].

**TABLE 1:** Risk predictors of lower back pain.

Physical	Psychological	Social
Low levels of physical activity	Anxiety, stress and depression	Smoking
Obesity	Fear avoidance beliefs	Education
Trauma: nociception, inflammation	Catastrophising (Irrational belief that something e.g., LBP, is worse than it is)	Poor work satisfaction
Previous episode of back pain	Job control and satisfaction	High physical work loads

**TABLE 2:** Management of Acute LBP < 6 weeks.

First line treatment for routine use	Second line or adjunctive treatment option	Not recommended
Advice to remain active	Non‐steroidal anti‐inflammatory drugs	Paracetamol
Education	Spinal manipulation, massage, acupuncture	Epidural glucocorticoid injection
	Opioids (limited use, selected patients)	
	Superficial heat	

**TABLE 3:** Management of Chronic LBP > 12 weeks.

First line treatment for routine use	Second line or adjunctive treatment option	Not recommended
Advice to remain active	Non‐steroidal anti‐inflammatory drugs	Paracetamol
Education	Spinal manipulation, massage, acupuncture	Systemic glucocorticoids
Exercise therapy	Mindfulness‐based stress reduction	Superficial heat
Cognitive behavioural therapy (CBT)	Interdisciplinary rehabilitation	
	Opioids (limited use, selected patients)	

The aim of this study was to determine the prevalence, impact, and understanding of LBP amongst final year dental students. The primary objectives were to determine the:

- Prevalence of LBP amongst final year undergraduate dental students
- Impact of LBP on recreational and professional activities
- Understanding of LBP amongst final year undergraduate dental students.

A secondary objective was to inform curricula development in the establishment of a novel undergraduate well‐being lecture series.

## 3 Materials and Methods

Ethical approval was granted by the UK university. A self‐administered online questionnaire was considered to be the most appropriate method of surveying the target population to address the objectives of the study. An anonymous, novel survey tool was designed using Survey Monkey.

Item generation was conducted following literature review and focus group sessions held with specialists and educators in physiotherapy, dentistry, and methodological experts. Concepts for exploration of the research question were defined, including factors affecting awareness of LBP, its prevention, and management. Item reduction was completed to restrict the questionnaire length and to minimise responder burden.

Quantitative data was gathered using mixed item formats and Likert scales. Closed‐ended questions included binary and dichotomous formats, which are exhaustive and mutually exclusive. Qualitative data was gathered using free text questions, allowing evaluation of the opinions of responders. The questionnaire was limited in length to 10 questions. Demographic data was not gathered as it was considered irrelevant to the research question, leading to responder bias or a suboptimal response rate if students had concerns that they might be identified. The single exception was the gathering of binary gender data.

The tool was pre‐tested via a face‐to‐face feedback discussion with clinicians, and subsequently piloted by a convenience sample of school staff (dentists and dental care professionals, *n* = 55) attending a lower back pain CPD lecture delivered by the undergraduate investigator who is a qualified physiotherapist. Impromptu written and verbal feedback was encouraged, which informed further development of the tool. Following consideration of pilot feedback, the tool was edited and re‐piloted on a convenience sample of attendees to a postgraduate study day. No further adjustments were necessary following the second pilot.

The target population included 100% of final year dental students at the UK School of Dentistry (*n* = 77). This consisted of dental students from undergraduate (*n* = 63 students) and graduate entry programmes (*n* = 14 students). Female students accounted for 65% (*n* = 50) of the population. Inviting 100% of the population negated the need for sampling. Participation was voluntary; hence it was important to engage the interest of responders at an opportune time in a busy year. The survey was administered prior to a careers evening event, to maximise response rate and engage interest. Students were invited to opt in and to complete the survey via their mobile phones or laptop devices. Once the survey had been completed by students who chose to opt in, all attendees received teaching with regard to the prevention and management of LBP, which was delivered by the undergraduate investigator to appropriately address any questions that arose from the audience. There was no pre‐notification of the survey or LBP lecture; hence, there was no incentive to revise the content in advance. Students who were absent on the evening were emailed a link for survey completion and an invitation to participate online via the same link within 24 h. The survey responses were not viewed until the survey period had closed at the end of this 24‐h period, thereby preventing any possibility of identifying absent students. No IP data was gathered; therefore, participation was truly anonymous. Data was exported into Excel and NVivo software for analysis.

## 4 Results

There was an 86% (*n* = 66) response rate and no missing item data within the responses. A majority of responders were female (70%, *n* = 46).

### 4.1 Prevalence of Lower Back Pain

LBP was a common experience for responders, with 58% (*n* = 38) reported to have suffered in the preceding 6 months. There was an increased incidence amongst female students, with 65% (*n* = 30) experiencing LBP compared to 37% of males (*n* = 7).

### 4.2 Impact of Lower Back Pain

Of the responders, 14% (*n* = 9) had accessed professional care for their LBP, 78% being female (*n* = 7) and 11% male (*n* = 1). Almost a third, 29% (*n* = 11) of responders reported that LBP had an impact on their recreational and professional activity. The average pain intensity score on visual analogue scales amongst responders was 37%.

### 4.3 Understanding of Lower Back Pain

**FIGURE 1:**
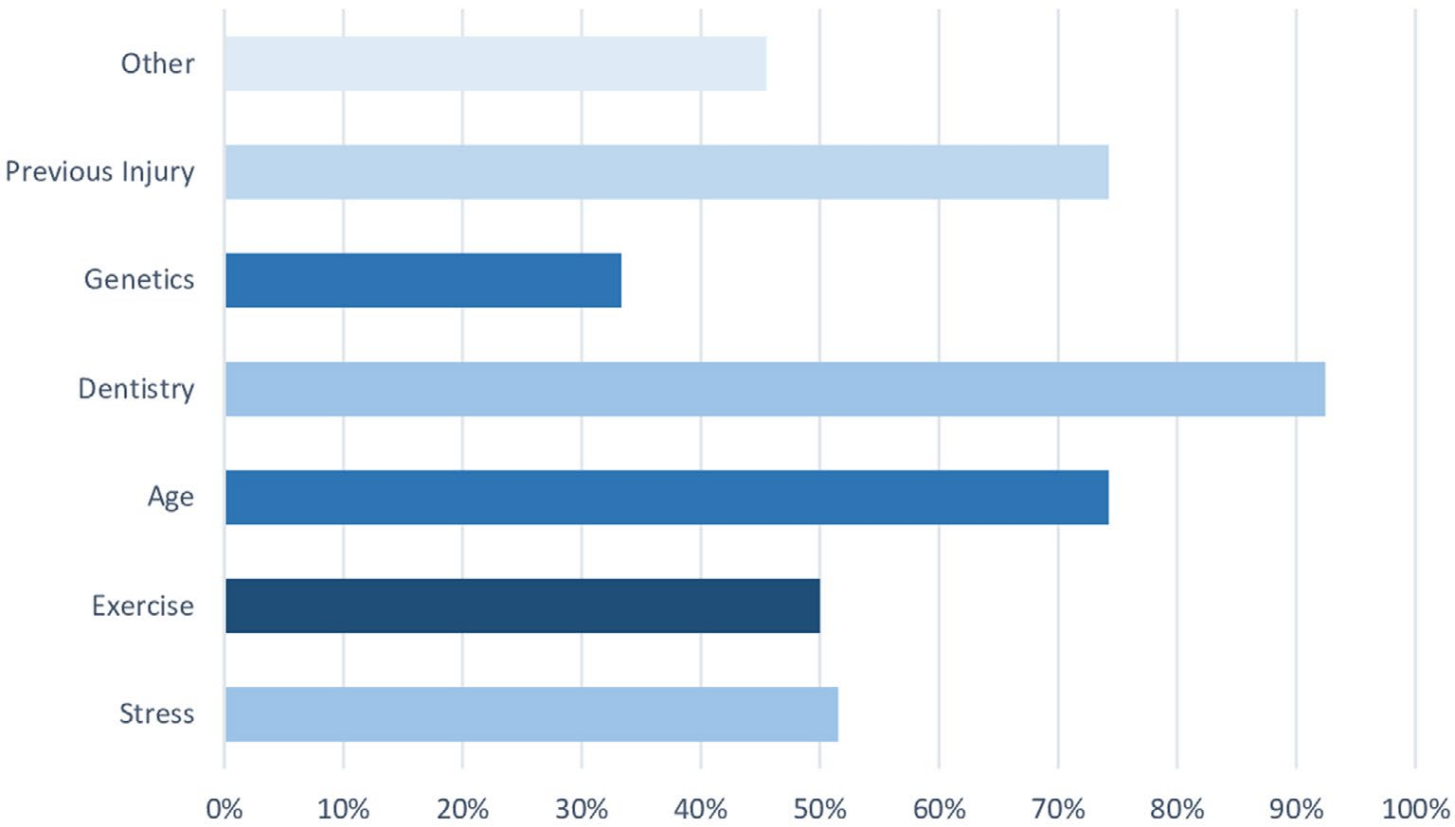
Final year student perceptions of factors predisposing them to developing lower back pain.

To gauge students' understanding of LBP, responders were asked to choose as many items as they wished from a list of predisposing risk factors to developing lower back pain. Figure 1 illustrates the results of this. A majority of students, 92% (*n* = 61), deemed ‘Dentistry’ to be the greatest risk factor for developing LBP. Other factors believed to be associated with worsening LBP were ‘previous injury’ 74% (*n* = 49), ‘age’ 74% (*n* = 49), ‘stress’ 52% (*n* = 34) and ‘exercise’ 50% (*n* = 33). Two students (3%) perceived that general bad posture could result in LBP, accounting for ‘other’ responses.

Participants were asked questions pertaining to their beliefs regarding management strategies for LBP. Table 4 demonstrates the results of this. As shown, over half, 53% (*n* = 35) of responders believed LBP would not resolve spontaneously without treatment. Fewer, 21% (*n* = 14), believed bed rest to be most effective in alleviating symptoms of LBP, whilst a majority refuted this. A majority, 88% (*n* = 58) of responders believed that maintaining correct posture during dental procedures was key to preventing LBP.

**TABLE 4:** Surveyed knowledge on management of lower back pain amongst 5th year BDS undergraduates.

Do you believe that lower back pain usually resolves by itself, whether or not, treatment is sought?	Is bed rest the most effective remedy to alleviate LBP?	Is correct posture during dental procedures the most effective way of preventing LBP?
14 (21%)	14 (21%)	58 (88%)
35 (53%)	36 (55%)	3 (5%)
17 (26%)	16 (24%)	5 (8%)

Students were asked how confident they would be managing their own LBP; (0 = not confident and 100 being very confident). The mean score for all responders was 39/100. Almost all, 98% (*n* = 65), responders believed that dental students should have an understanding of LBP in preparation for their future careers and supported the inclusion of relevant teaching with regard to risk, prevention, and management in undergraduate curricula.

### 4.4 Exercise Undertaken by Dental Cohort and Perceived Fitness Levels

**FIGURE 2:**
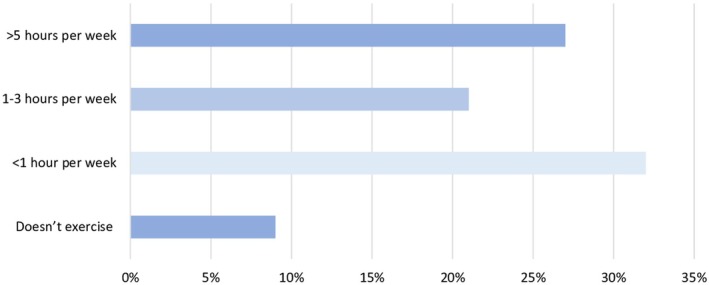
Average weekly self‐reported exercise of final year dental students.

Figure 2 illustrates the average weekly self‐reported exercise of the cohort. ‘Average’ to ‘good’ levels of fitness were reported by 92% (*n* = 62) of responders, with 48% (*n* = 32) undertaking more than 1 h of exercise per week.

Responders were asked to provide free text responses to a single open question; ‘do you have any further thoughts to share about lower back pain and your career as a health professional?’ Responses were thematically analysed and categorised as (1) requesting funding for ergonomic equipment such as loupes and saddle chairs (2) expressing concern that dentistry worsens back pain.

‘Would like to use saddle chairs more often to correct posture. Worried career will be short lived if back pain not considered important’.

‘Back pain for ten years. Saw physio last week NHS and one privately. I have had bad posture since childhood dentistry likely making it worse’.

‘It's very helpful to become aware of it while still studying so we can take steps to avoid it early in our careers’.

‘I think dental schools should consider funding/contributing to provision of loupes to aid with posture and minimise risk of back pain in future dentists’.

‘I am concerned that dentistry might worsen lower back pain’.

## 5 Discussion

This study was designed to assess the prevalence, impact, and understanding of LBP amongst a graduating cohort of dental students. The good response rate achieved indicates that the participating population were engaged with learning about well‐being. The cohort is likely to be generalisable across other UK dental schools who present with similar demographics as reported here. A minority of eligible students did not respond, and their views have not been accounted for. It is possible that these students did not engage if they have not previously experienced LBP and have little interest in the subject at present. It is also possible that the opposite is true and that these students have experienced distressing LBP that discouraged their participation.

A majority of the eligible cohort (65%) and responders (69%) were female. Similar gender demographics (63% female) were reported in a similar earlier study [4]. Despite females being more likely to report a higher average pain intensity and prevalence of LBP compared to their male counterparts, this gender balance is in line with that of the national undergraduate student body [4, 8, 9, 10].

This study revealed a high 6‐month prevalence of musculoskeletal LBP (58%) amongst undergraduate students in their final year. Whilst this is perhaps surprising, this finding is in line with similar studies exploring the prevalence of LBP. A cross‐sectional study by Viray et al. revealed the prevalence amongst undergraduate dental students to be 53% [4]. Similarly, Pejcic et al. found the prevalence to be even higher at 81.8% during undergraduate training [17]. Further research is necessary to determine whether this apparent increase in LBP is significant and generalisable across the UK and further afield; however, the experiences of dental students are likely to be similar by final year irrespective of their place of study. The results of this study corroborate with international findings; in a Californian dental school, Rising et al. found that more than 70% of dental students of both sexes reported experiencing musculoskeletal pain by their third year [18]. In the pilot study conducted for this paper, 64% of responders had experienced LBP within 6 months; these responders were qualified dentists and dental care professionals, and it is of interest for further research to determine whether LBP increases exponentially throughout a career in dentistry or whether individualised risk factors remain the most reliable predictors for those who suffer. LBP prevalence across years 1 to 5 of an undergraduate programme reportedly increases and peaks in the final year [4, 8]. No long‐term studies could be found that investigated the prevalence or impact of LBP of dentists commencing prior to graduation as they progressed through their careers.

The impact of LBP on dental students who have suffered is notable, with 14% of the cohort having sought treatment from a healthcare professional and 29% reducing their professional or recreational activities within the 6 months prior to the survey. The results from the pilot study suggested qualified dentists might be more willing to seek professional help (27%); however, interestingly they reported less of an impact on work and leisure activities (19%). It is possible that qualified dentists have better access to resources to seek aid, and that those who have suffered LBP may have already reduced working hours in an effort to manage their pain. Dental students are required to attend scheduled undergraduate teaching that may not offer flexibility; however, they are perhaps less likely to undertake prolonged sedentary stationary positions as frequently as their qualified counterparts. Undergraduate and qualified dental professionals may lack education on musculoskeletal disorders and have a poor understanding of the prevention and management of LBP that might contribute to this experience [4, 5, 6, 7].

Understanding of LBP was assessed using questions carefully selected following a review of the limited validated evidence base, and with a robust methodological approach to survey design. The specificity of the questionnaire was designed to inform curricula development. Although 87% of students believed a ‘correct’ posture was the most effective way to prevent LBP, evidence shows this belief system may have negative influences on dental professionals' lower back health [19]. Despite the former hypothesis, the evidence linking poor posture and LBP is conflicting. Some studies have revealed no evidence to link poor posture and LBP [15, 19, 20, 21, 22, 23]. In fact, a systematic review found poor posture can be protective and teaching a ‘correct’ sitting or standing posture can lead to the person using guarded movement patterns, fearing incorrect positions, and lowering their self‐efficacy, which may lead to chronic and persistent pain [15, 19, 24].

An umbrella review into risk and preventative measures of musculoskeletal disorders in the dentistry environment found that it is ‘static’ and often ‘awkward’ postures that have a key role in the aetiology of musculoskeletal disorders as well as repetitive movements and muscle imbalances. The maintenance of a neutral balanced posture with frequent alternation between different positions was found to be protective. The importance of a modern and ergonomic workstation was highlighted as well as the value of stretching at the end of a work session [25].

Poor understanding of LBP risk factors, prevention and management techniques may lead to poor recovery expectations, catastrophism, increased pain levels and in some circumstances chronic and persistent pain [13, 15]. The current study found 9% of students did not participate in any weekly exercise, 50% suggested exercise may make LBP worse, and 20% advocated bed rest to be the most effective remedy for LBP. Evidence suggests recovery from LBP is more effective when patients are encouraged to stay active as exercise should be the first line management and prevention strategy for LBP [15, 16, 19, 26]. Over half of responders believe LBP requires treatment to recover. In fact, 90% of LBP is non‐specific, the majority recovers in 4–6 weeks with or without treatment [9]. Lack of knowledge and poor recovery expectations may contribute to the high pain intensity and prevalence evident in these findings.

A high self‐reported good to average fitness (94%) was evident, with a third adhering to the NHS guidance of 150 min of weekly exercise [27]. Notably, descriptors of levels of fitness were not provided, and the findings are subjective. Similar results were found in other studies, with higher self‐reported fitness levels in more senior dental years [4, 8]. Dental students participating in 90 min of aerobic cardiovascular exercise over 3 days a week reportedly experienced improved lower back health [8]. This concurs with current research in the general population, which found strengthening, stretching, or aerobic exercises performed 2–3 times weekly can help prevent LBP [28]. Also, a number of systematic reviews report higher levels of fitness correlate with lower intensity and prevalence of LBP [16, 26, 28]. Despite the high levels of self‐reported fitness, LBP prevalence and pain intensity peaked in the final year [4, 8]. This may be due to students inaccurately self‐reporting fitness levels, with more reliable results being sought through physical assessments or validated pain and fitness scales such as the Nordic lower back pain questionnaire or the CLINIMEX Aerobic Fitness Questionnaire [29, 30]. However, as other studies reported similar results, perhaps confounding factors are increasing LBP in final year dental students.

A longitudinal study has identified four key LBP risk factors including obesity, smoking, depression and stress [31]. Final year students are reported to experience the highest stress levels amongst the undergraduate dental cohort, affecting their physical and psychological well‐being [32]. Therefore, it is possible that LBP may peak in the final year of dental school. This is worthy of consideration by dental educators and those tasked with programme design, and promotes the need for a shift away from a biomedical model of education focussed on ergonomics and posture, to a biopsychosocial model of education incorporating exercise and mindfulness [16, 26]. This model may help reduce the high impact LBP has on graduating dentists and throughout their career.

In this study, there was strong support (98%) for incorporation of LBP education into the dental curricula. Indeed, other authors have emphasised the desirability of new educational models for dental educators to reduce work‐related musculoskeletal disorders [33]. The value of incorporation of back pain prevention programmes into the undergraduate curriculum has been shown. An evaluation after a 10‐year implementation of a back pain prevention programme into the faculty of dentistry in Strasbourg, France, revealed a positive preventive effect on both the prevalence of back pain and back pain intensity [34].

It could be speculated that possible limitations to this study are that the researcher could not fully ascertain that the responders had fully understood the survey questions in their absence; although the piloting exercise helped to negate this. It was also not possible to ensure that responders were allowed a sufficient amount of time to provide detailed answers to the free‐text questions, and completion on a mobile phone device may have discouraged lengthy answers. Bias was also introduced because of the self‐reporting subjective nature of the survey, especially, for example, when considering fitness levels as previously alluded to. It was felt, however, that the opportunity to maximise response rate with a self‐report survey at a convenient time would offset this potential disadvantage.

It could be postulated that further weaknesses of the study include the omission of demographic variables such as age, height, weight, BMI, and socioeconomic status. The absence of demographic diversity analysis may limit the generalisability of the findings. Although it may have been interesting to analyse how these variables affected the prevalence of LBP, it was felt that this was irrelevant to the main research questions; excluding them would ensure true anonymity and likely increase response rate.

## 6 Conclusion

The findings of this single‐centre study indicate that LBP has a high prevalence and impact amongst final year UK dental students. The results have informed local curricula development, and this best practice is shared to encourage dental educators to consider the same. Further research is desirable into the value and effectiveness of ergonomic assessment and equipment provision for dental undergraduates. The results of this survey have been disseminated to the undergraduate dental population via BDJ Student [35]. A recorded webinar and practical session have been designed for incorporation into undergraduate curricula to support a biopsychosocial approach to education, prevention, and management. Further research to include qualitative methods regarding the effectiveness of this educational support and self‐care advice is needed to confirm the success of a biopsychosocial approach to LBP in dental students.

## Author Contributions

**Samuel Cope:** literature review, data collection and analysis, write up. **Elizabeth Willasey:** update literature review, write up. **Daniel Dadnam:** aided in data collection and analysis and write up. **Laura Gartshore:** study design, ethical approval, research supervisor, write up.

## Ethics Statement

Ethical approval was granted by the University of Liverpool.

## Conflicts of Interest

The authors declare no conflicts of interest.

## Data Availability

The data that support the findings of this study are available from the corresponding author upon reasonable request.
